# Historical contingency in the evolution of antibiotic resistance after decades of relaxed selection

**DOI:** 10.1371/journal.pbio.3000397

**Published:** 2019-10-23

**Authors:** Kyle J. Card, Thomas LaBar, Jasper B. Gomez, Richard E. Lenski

**Affiliations:** 1 BEACON Center for the Study of Evolution in Action, Michigan State University, East Lansing, Michigan, United States of America; 2 Department of Microbiology and Molecular Genetics, Michigan State University, East Lansing, Michigan, United States of America; 3 Program in Ecology, Evolutionary Biology, and Behavior, Michigan State University, East Lansing, Michigan, United States of America; 4 Department of Molecular and Cellular Biology, Harvard University, Cambridge, Massachusetts, United States of America; 5 Biomedical Laboratory Diagnostics Program, Michigan State University, East Lansing, Michigan, United States of America; Biological Research Center, HUNGARY

## Abstract

Populations often encounter changed environments that remove selection for the maintenance of particular phenotypic traits. The resulting genetic decay of those traits under relaxed selection reduces an organism’s fitness in its prior environment. However, whether and how such decay alters the subsequent evolvability of a population upon restoration of selection for a previously diminished trait is not well understood. We addressed this question using *Escherichia coli* strains from the long-term evolution experiment (LTEE) that independently evolved for multiple decades in the absence of antibiotics. We first confirmed that these derived strains are typically more sensitive to various antibiotics than their common ancestor. We then subjected the ancestral and derived strains to various concentrations of these drugs to examine their potential to evolve increased resistance. We found that evolvability was idiosyncratic with respect to initial genotype; that is, the derived strains did not generally compensate for their greater susceptibility by “catching up” to the resistance level of the ancestor. Instead, the capacity to evolve increased resistance was constrained in some backgrounds, implying that evolvability depended upon prior mutations in a historically contingent fashion. We further subjected a time series of clones from one LTEE population to tetracycline and determined that an evolutionary constraint arose early in that population, corroborating the role of contingency. In summary, relaxed selection not only can drive populations to increased antibiotic susceptibility, but it can also affect the subsequent evolvability of antibiotic resistance in an unpredictable manner. This conclusion has potential implications for public health, and it underscores the need to consider the genetic context of pathogens when designing drug-treatment strategies.

## Abstract 2

Strains of Escherichia coli from the long-term evolution experiment (LTEE) have independently evolved for multiple decades and tens of thousands of generations in the absence of antibiotics; this study shows that evolution and diversification of a single bacterial species in an antibiotic-free environment can render the subsequent evolution of resistance unpredictable.

## Introduction

A population may encounter an environmental change that removes or reduces a selective pressure that was previously important for the maintenance of a trait [1,2]. Adaptation to the new environment can therefore affect an organism’s fitness in its prior environment. These correlated responses may lead to the functional decay of unused traits over time or, conversely, their maintenance despite relaxed selection [2]. However, the evolutionary processes driving these responses are often hard to disentangle because one must rely on retrospective studies and historical inference.

By contrast, evolution experiments with microorganisms provide a powerful approach to study correlated responses. Microbes often have large population sizes and fast generations, and they are amenable to freezing and revival. One can therefore observe evolution in action, directly compare ancestral and derived forms, and simultaneously assess adaptation to one environment and quantify correlated fitness responses in another. Accordingly, numerous studies with bacteria [3–9], viruses [10–15], and yeast [16–18] have found that fitness trade-offs between environments are common.

Trade-offs are often caused by antagonistic pleiotropy, which occurs when a mutation that is beneficial in one environment is deleterious in another. This process can have important public health consequences when antibiotic-resistance mutations or acquired resistance genes impose costs on bacterial growth and competitiveness relative to their sensitive counterparts in the absence of drugs [19,20]. Previous studies have shown that pleiotropic fitness costs are widespread among resistance determinants to diverse drug classes [21–24], although their magnitudes are variable and may also depend on the genetic background [20,25–27].

Given that antibiotic-resistance mutations and genes commonly impose fitness costs, one would expect that resistance should decline over time in the absence of antibiotic exposure. However, compensatory evolution often reduces or eliminates these trade-offs [22,23,28,29]. Adaptive trends during compensatory evolution have been studied using a number of *E*. *coli* mutants resistant to the drug rifampicin [30]. That study found that the mutants were generally less fit than their sensitive progenitors in a permissive antibiotic-free environment; moreover, the compensatory effects of subsequent beneficial mutations were greater when the resistance was more costly. Thus, compensation exhibited a pattern of diminishing-returns adaptation in that study.

Even when bacteria have no known history of exposure to antibiotics, they may have low-level resistance to some drugs because of intrinsic structural or functional features, including their cell envelope and efflux pumps [31]. As a consequence, intrinsic resistance may decline in the absence of drug exposure if relevant genes accumulate mutations either by selection or drift in permissive environments [6].

A recent study used the *E*. *coli* long-term evolution experiment (LTEE), and antibiotic resistance as a model trait, to study changes in an organism’s capacity to tolerate environmental stresses when it evolves for a long period in the absence of those stresses [32]. In the LTEE, 12 replicate populations were founded from a common ancestor and have been propagated daily for over 30 years in a medium without antibiotics [33,34]. In particular, Lamrabet and colleagues measured changes in mostly low-level intrinsic resistance between ancestral and derived strains isolated from each population after generations 2,000 and 50,000. They found that derived strains were usually more susceptible to most antibiotics than their ancestor, and from multiple lines of evidence they inferred that these losses of intrinsic resistance resulted primarily from pleiotropic side effects of beneficial mutations that arose during the LTEE.

Although the lineage leading to the LTEE ancestor has no known history of exposure to industrially manufactured antibiotics (except streptomycin), it might nevertheless have a history of exposure to similar compounds produced by competitors and to host bile salts. Adaptations that provide resistance to these other stressors, such as those involving the cell envelope and efflux pumps [35], often confer intrinsic resistance to antibiotics [31]. Thus, even the low-level resistance of the ancestor might reflect this prior natural history, and selection was relaxed on these traits in the LTEE environment.

**Fig 1:**
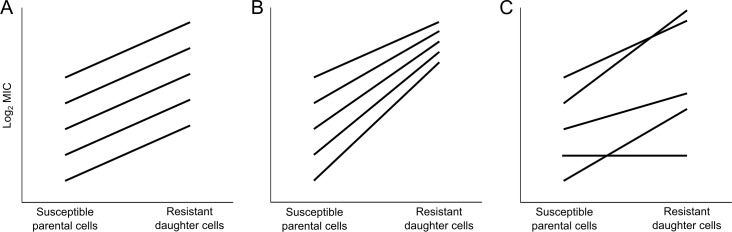
Schematic illustration of the evolvability of antibiotic resistance under three scenarios. A strain’s evolvability is defined operationally as the maximum increase in resistance from an initially susceptible genotype during one round of drug selection. (A) Null model, with no effect of genetic background on evolvability. (B) Diminishing-returns model, such that backgrounds with low initial resistance are more evolvable than backgrounds that are initially more resistant. (C) Idiosyncratic-effects model, in which evolvability varies among genetic backgrounds but is uncorrelated with their initial level of resistance. MIC, minimum inhibitory concentration.

Taken together, the experimental evolution studies described above have two contrasting implications relevant for medicine and public health. First, resistance to antibiotics (including even low-level intrinsic resistance) may decline in the absence of drug exposure. Second, evolution can often compensate for deleterious side effects of mutations, thereby facilitating the maintenance of evolved resistance. The question then arises how readily bacteria can overcome losses of antibiotic resistance that arose during periods of relaxed selection through subsequent evolution in the presence of drugs. In this study, we address this fundamental question by using the LTEE ancestor and derived strains isolated from four populations after 50,000 generations to examine how evolution in the absence of antibiotics affects the bacteria’s potential to evolve increased resistance when drugs are introduced. In so doing, we examine the role that genetic background plays in resistance evolvability (Fig 1). Does resistance evolution tend to follow a general trend of diminishing returns [30,36–38], such that derived strains that are initially more susceptible to a drug can increase their resistance disproportionately relative to their ancestor (Fig 1B)? Or is evolvability idiosyncratic with respect to prior evolutionary history [39,40], such that the relative gains in resistance are independent of a strain’s initial susceptibility (Fig 1C)?

Throughout this paper, we discuss how differences in genetic background may affect the evolvability of antibiotic resistance. This focus brings to mind the concept of epistasis, whereby the marginal effect of a particular mutation on some phenotype of interest depends on its interaction with another mutation or, more generally, the set of mutations that distinguish genetic backgrounds [38,41–47]. In the context of our study, epistasis could arise in at least two ways. First, mutations in the same target gene may confer different levels of resistance depending on other mutations that differ between backgrounds. Second, the physiological mechanisms and associated loci underlying resistance may differ across genetic backgrounds. It might seem unexpected that the genetic basis of resistance would differ among closely related backgrounds. However, as we will show, the initial levels of resistance vary among backgrounds, and the physiological mechanisms that allow cells to resist drugs may depend on their concentrations, such that the mechanisms used may also differ across backgrounds. Without a more precise mechanistic understanding at this stage of our work, we cannot distinguish between these forms of epistasis. More generally, we will use the term evolvability (rather than epistasis) because it emphasizes the consequences of these effects for antibiotic resistance.

We confirmed the finding of Lamrabet and colleagues [32] that the LTEE-derived strains had typically become more susceptible to antibiotics during relaxed selection. However, contrary to our expectation based on a diminishing-returns model, we discovered that these derived strains were usually no more evolvable (and sometimes less evolvable) than their ancestor when exposed to various antibiotics. Instead, idiosyncratic responses dominated over any diminishing-returns tendency, such that the capacity to evolve resistance was hampered on some LTEE-derived genetic backgrounds. These results indicate that evolution and diversification of a single bacterial species in a permissive environment can lead to unpredictable changes in the potential to evolve antibiotic resistance. Our work suggests that methods for predicting, at the strain level, a pathogen’s evolutionary potential should be developed in light of the global threat of antibiotic resistance. If successful, such methods could become an integral aspect of resistance surveillance and patient treatment.

## Results

### Antibiotic susceptibility profiles of the LTEE ancestral and derived clones

**Fig 2:**
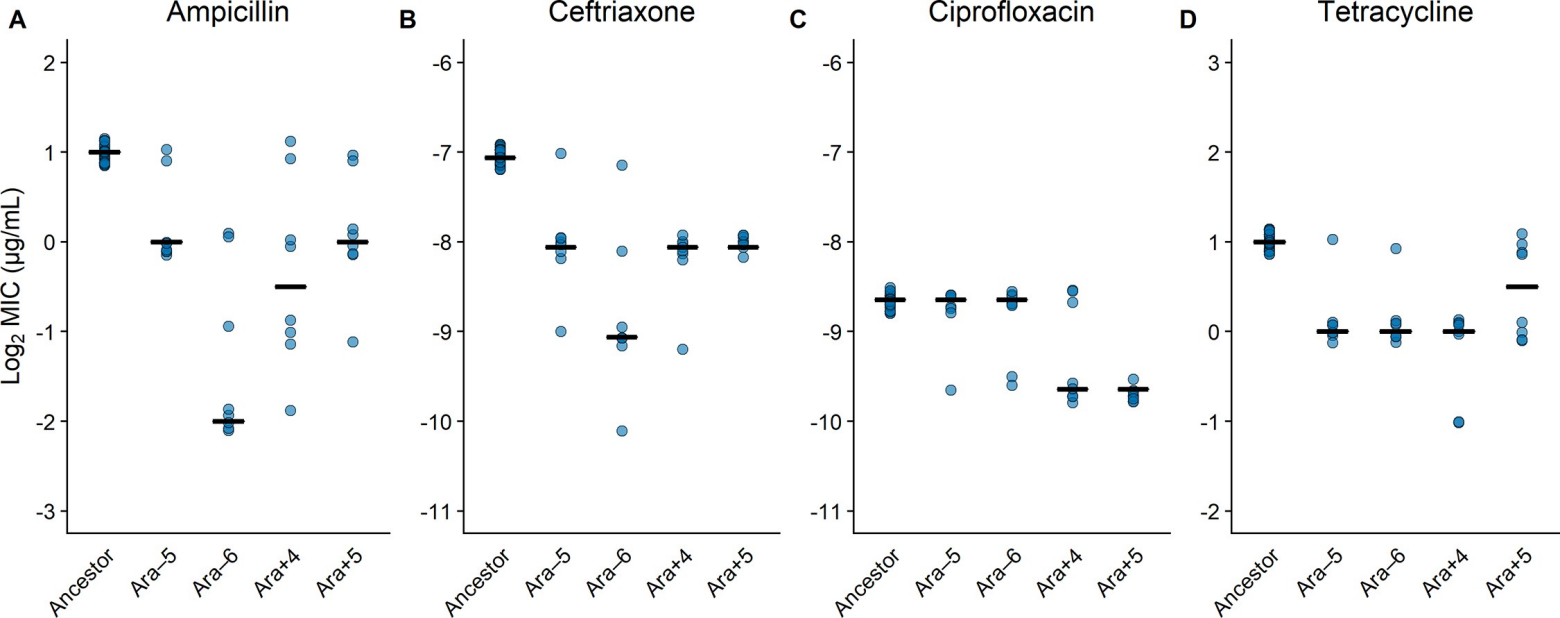
Intrinsic resistance usually declined over time in the absence of drug exposure. Comparison of the LTEE ancestor and four independently derived clones sampled after 50,000 generations for their susceptibilities to ampicillin, ceftriaxone, ciprofloxacin, and tetracycline (A–D). MICs are shown on a log_2_-transformed scale to reflect the fact that antibiotic concentrations were tested across a series of 2-fold dilutions. In each panel, points show values obtained from 32 and 8 replicate assays for the ancestor and derived strains, respectively. Horizontal bars show the median of the log_2_-transformed MIC values for each strain on each antibiotic. The absolute values of the concentrations shown on the y-axis differ among the four antibiotics, but the range is the same in each panel. The underlying data for this figure may be found here: https://datadryad.org/stash/dataset/doi:10.5061/dryad.g41hg96. LTEE, long-term evolution experiment; MIC, minimum inhibitory concentration.

Antibiotic susceptibility measurements were generally quite repeatable (Fig 2). For each antibiotic, all 32 independent ancestral replicate minimum inhibitory concentration (MIC) measurements were identical. Among the 16 sets of derived-clone replicates (4 clones × 4 antibiotics), the 8 replicate assays gave identical MICs in 2 cases (12.5%), they deviated minimally by a factor of 2 in 12 cases (75%), and in only 2 cases they deviated by a factor of 4 (12.5%). These results provide strong support for the use of our plate-based approach, as described in the Materials and methods and shown in S1 Fig, to quantify antibiotic susceptibility profiles.

### Changes in susceptibility under relaxed selection during the LTEE

For each antibiotic, we made 32 comparisons between the MICs of derived clones (4 clones × 8 replicates) against their paired and independently isolated ancestral clones. On balance, we observed increased susceptibility of the strains that evolved under relaxed selection (i.e., in the absence of antibiotics) during the LTEE, consistent with recently published results [32] (Fig 2). All four derived strains have increased sensitivity to ampicillin (Fig 2A), ceftriaxone (Fig 2B), and tetracycline (Fig 2D) relative to their common ancestor, and two of the derived strains were more susceptible to ciprofloxacin (Fig 2C). These trends toward lower resistance are well supported by trinomial tests, as described in the Materials and methods and as shown in Table 1 and S1 Table.

**Table 1:** Statistical analyses of declines in intrinsic resistance during relaxed selection of clones sampled at generation 50,000 of the LTEE.

Antibiotic	χ^2^	*p*
Ampicillin	40.06	<0.0001
Ceftriaxone	45.33	<0.0001
Ciprofloxacin	27.15	0.0007
Tetracycline	41.40	<0.0001

Analyses were performed using Fisher’s combined probability method (df = 8) for multiple independent tests of the same hypothesis, with an underlying trinomial distribution for the null hypothesis.

Abbreviation: LTEE, long-term evolution experiment

### Evolvability profiles of the ancestor and derived clones

Next, we examined how the prior history of relaxed selection affected the evolvability of antibiotic resistance in the different genetic backgrounds. To address this question, we selected mutants of the ancestral and LTEE-derived strains that survived and grew sufficiently to form colonies at higher concentrations of the four antibiotics than their corresponding parental strains (S1 Fig). As described in the Materials and methods, we operationally define evolvability as the maximum observed increase in antibiotic resistance from an initially susceptible genotype during one round of drug selection (Fig 1).

Evolvability measurements tended to be more variable than the MIC measurements. We examined the evolvability of 128 independent ancestral clones across the four antibiotics. There were 73 cases (57%) in which these measurements corresponded to the median for that antibiotic, 49 cases (38.3%) in which they differed by a factor of 2, and 6 cases (4.7%) in which they differed by a factor of 4. Likewise, among the 16 sets of replicates for the LTEE-derived clones, the 8 assays varied by a factor of 2 in 12 cases (75%) and by a factor of 4 in 4 other cases (25%). The greater variation in evolvability measurements in comparison with MIC values among replicate assays is expected given the stochastic appearance of mutations in replicate cultures [48]. Also, increased resistance can occur through multiple mutational paths [49,50], and those mutations affecting one mechanism might confer greater resistance evolvability relative to mutations affecting some other mechanism.

### Effects of genetic background on the evolvability of resistance

**Fig 3:**
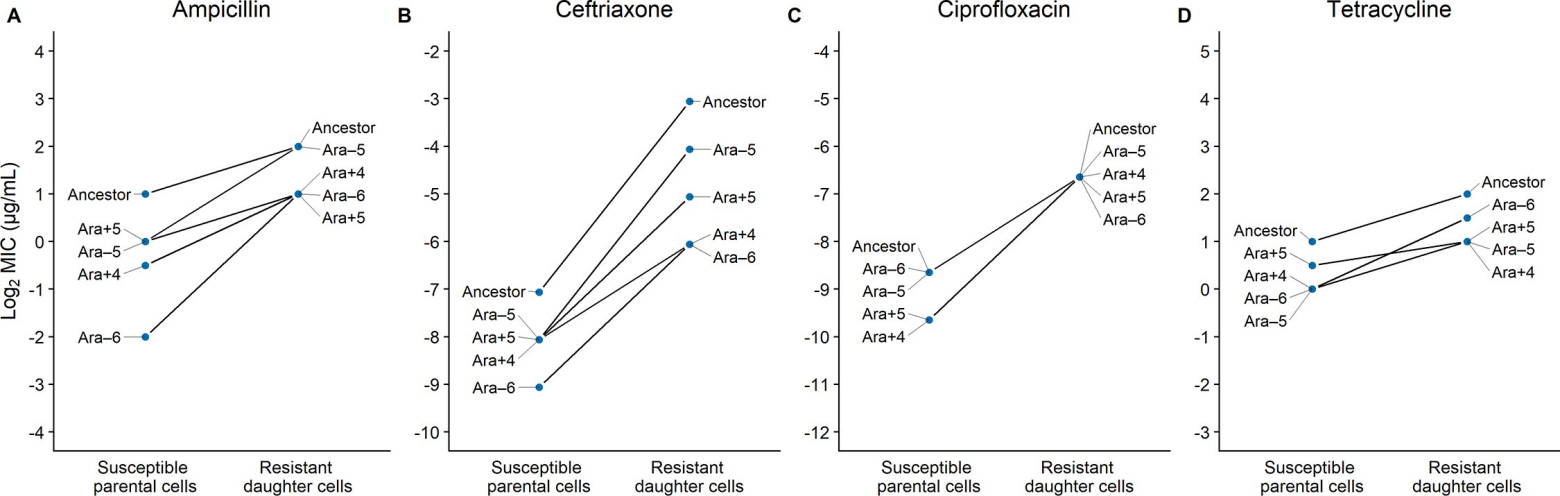
Genetic background affects the evolvability of LTEE lines exposed to antibiotics. Lines joining susceptible parental strains with their daughter mutants show the increases in resistance during one round of selection with ampicillin, ceftriaxone, ciprofloxacin, and tetracycline (A–D). If the slope of a derived strain is greater than that of the ancestor, then it has greater evolvability; and vice versa. Median MICs are shown on a log_2_-transformed scale to reflect the fact that antibiotic concentrations were tested across a series of 2-fold dilutions. The y-axis ranges for the four drugs have been scaled to the ceftriaxone environment, which had the largest gains in resistance between the susceptible parental cells and the resistant daughter cells. The underlying data for this figure may be found here: https://datadryad.org/stash/dataset/doi:10.5061/dryad.g41hg96. LTEE, long-term evolution experiment; MIC, minimum inhibitory concentration.

We examined the possibility of two broad patterns of genetic-background effects with respect to resistance evolvability in our study. First, we asked whether evolvability followed a trend of diminishing returns, such that the more susceptible LTEE-derived genetic backgrounds generally produced mutants with proportionally greater gains in resistance than the ancestor. Both the ancestral and derived strains evolved resistance to varying degrees (Fig 3). The evolutionary potential of two of the four derived clones (Ara–5 and Ara–6) was noticeably greater relative to their ancestor in the ampicillin environment (Fig 3A), but there were no clear instances of similar trends in the three other drug environments (Fig 3B–3D).

Overall, there was no statistical support for the diminishing-returns trend, despite the visual impression for the ampicillin treatment. We compared each derived strain’s evolutionary potential with its paired ancestor in the four drug environments. We used trinomial tests to quantify the likelihood that each derived strain’s evolvability was greater than its ancestral counterpart when tested against the null hypothesis of equally frequent changes in either direction, after taking into account the many numerical ties [51]. Although the capacity of the derived Ara–5 clone to evolve increased resistance was significantly greater than its ancestor when considered in isolation (S2 Table), it was marginally nonsignificant when we examined overall trends for each antibiotic (Table 2) using a meta-analysis approach [52,53].

**Table 2:** Statistical analyses of diminishing-returns trends in resistance evolvability of clones sampled at generation 50,000 of the LTEE.

Antibiotic	χ^2^	*p*
Ampicillin	14.63	0.0668
Ceftriaxone	0.18	1
Ciprofloxacin	7.88	0.4456
Tetracycline	5.97	0.6511

Analyses were performed using Fisher’s combined probability method (df = 8) for multiple independent tests of the same hypothesis, with an underlying trinomial distribution for the null hypothesis.

Abbreviation: LTEE, long-term evolution experiment

We then asked whether the proportional resistance gains when exposed to the antibiotics were idiosyncratic among LTEE lines. For example, the capacity to evolve ceftriaxone resistance appeared to be reduced among three LTEE-derived backgrounds (Ara+5, Ara–6, and especially Ara+4) relative to their common ancestor (Fig 3B). Similarly, the evolvability of the Ara+5 background with respect to tetracycline appears to be constrained (Fig 3D). Indeed, this latter case was the only one in which the mutants of a strain systematically achieved a lower level of resistance than did the mutants of other strains that were initially more susceptible (indicated by the crossing lines in Fig 3D). These idiosyncratic tendencies are statistically well supported by Kruskal-Wallis tests. For both ceftriaxone and tetracycline, these tests reject the null hypothesis of homogeneity in proportional resistance increases across the different genetic backgrounds (Table 3).

**Table 3:** Statistical analyses of idiosyncratic patterns in resistance evolvability of clones sampled at generation 50,000 of the LTEE.

Antibiotic	χ^2^	*p*
Ampicillin	9.19	0.0566
Ceftriaxone	23.45	0.0001
Ciprofloxacin	7.59	0.1077
Tetracycline	10.18	0.0376

Analyses were performed using a Kruskal-Wallis one-way nonparametric ANOVA (df = 4).

Abbreviation: LTEE, long-term evolution experiment

**Fig 4:**
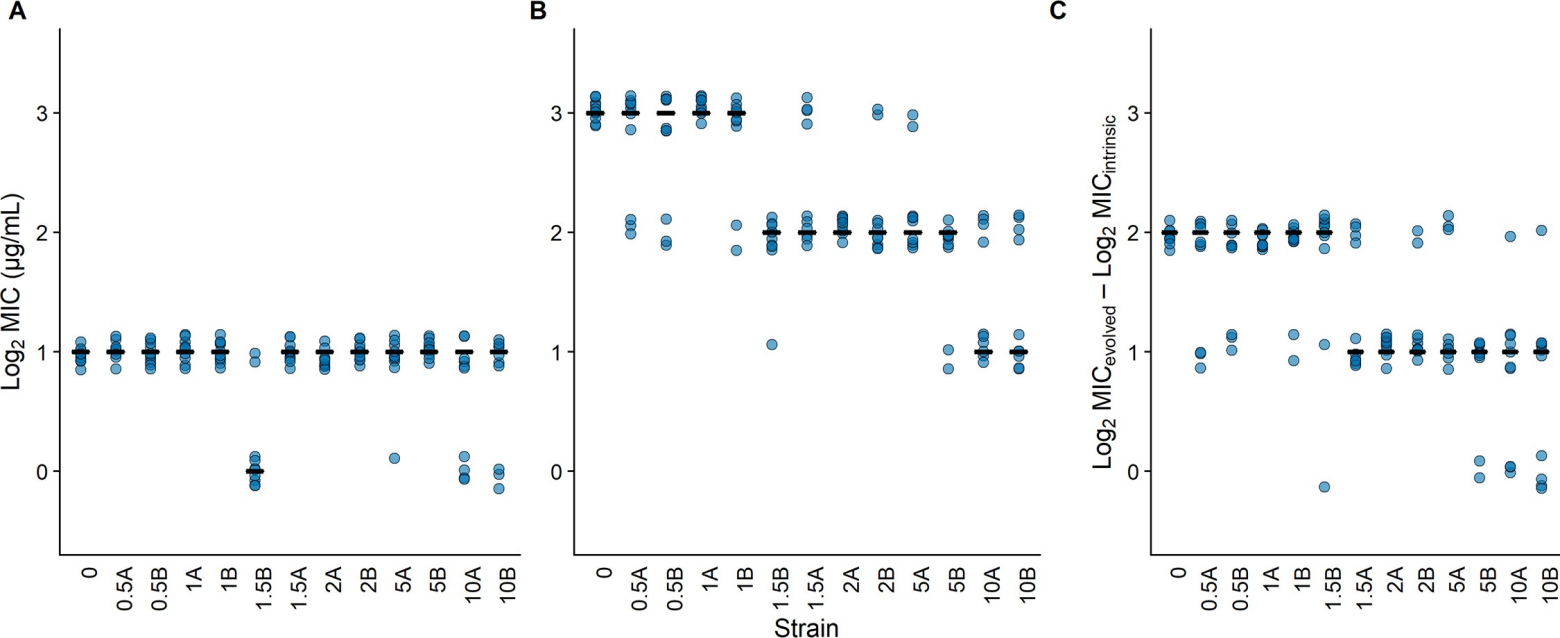
Capacity to evolve tetracycline resistance was diminished early in one LTEE lineage. (A) Comparison of the intrinsic tetracycline resistance of the ancestor and a time series of derived strains isolated from the Ara+5 population. Strains are ordered by their time of isolation. The strain identifiers begin with a number corresponding to the generation (in thousands) of their isolation, followed by an arbitrary letter; strains 2A and 2B, for example, are two clones isolated at generation 2,000 of the LTEE. (B) Comparison of the evolved resistance levels after one round of drug selection, based on the MICs of the mutant daughter cells derived from the corresponding parental strains. MICs are shown on a log_2_-transformed scale to reflect the fact that the concentrations of antibiotics were tested across a series of 2-fold dilutions. (C) Evolvability is quantified for each strain as the difference in the log_2_-transformed MICs of the parental strain and its corresponding daughter mutant. Points show 10 independent replicates per strain. Horizontal bars show the median log_2_-transformed MICs (A, B) for 10 replicate assays and the evolvability (C) based on the corresponding 10 paired differences. The underlying data for this figure may be found here: https://datadryad.org/stash/dataset/doi:10.5061/dryad.g41hg96. LTEE, long-term evolution experiment; MIC, minimum inhibitory concentration.

Given these idiosyncratic effects of genetic background, we chose to examine one of the cases in greater detail. In particular, we asked when the evolvability of the Ara+5 background declined with respect to tetracycline. To address this question, we examined clones isolated during this population’s early history and tested whether they had lost their capacity to evolve tetracycline resistance during a single exposure, to an extent commensurate with the ancestral strain’s evolvability. As shown in Fig 4, the reduced evolvability was evident in all of the clones isolated from generation 2,000 onward as well as in one of two clones isolated at generation 1,500. With one exception, all of the LTEE-derived parental backgrounds across this time series had the same MIC value as the ancestor (Fig 4A). However, the daughter mutants from the later-generation Ara+5 genetic backgrounds had progressively lower levels of tetracycline resistance (Fig 4B), which when coupled with the same initial resistance level indicates they had become less evolvable in this respect (Fig 4C). A Kruskal-Wallis test decisively rejects the null hypothesis of equal evolvabilities across the entire set of clones (χ^2^ = 67.89, df = 12, *p* < 0.0001), and Dunnett’s tests comparing the evolvability of each derived clone from LTEE population Ara+5 with that of the ancestor support the temporal break point described above (Table 4).

**Table 4:** Statistical analyses comparing tetracycline resistance evolvability of clones isolated from the Ara+5 population at different generations to the LTEE ancestor.

Strain	Difference	Lower 95% CI	Upper 95% CI	*p*
0.5A	−0.3	−0.9	0.3	0.6921
0.5B	−0.3	−0.9	0.3	0.6921
1A	0.0	−0.6	0.6	1
1B	−0.2	−0.8	0.4	0.9607
1.5B	−0.3	−0.9	0.3	0.6921
1.5A	−0.6	−1.2	0.0	0.0412
2A	−1.0	−1.6	−0.4	<0.0001
2B	−0.8	−1.4	−0.2	0.0021
5A	−0.7	−1.3	−0.1	0.0101
5B	−1.2	−1.8	−0.6	<0.0001
10A	−1.2	−1.8	−0.6	<0.0001
10B	−1.3	−1.9	−0.7	<0.0001

Analyses were performed using a Dunnett’s test. Strain identifiers begin with a number that corresponds to the generation (in thousands) of their isolation, followed by an arbitrary letter; strains 2A and 2B, for example, are two clones isolated at generation 2,000 of the LTEE.

Abbreviation: LTEE, long-term evolution experiment

### Multiple factors can contribute to differences in evolvability

The observed changes in evolvability of tetracycline resistance in the Ara+5 population could, in principle, reflect several factors, including differences among genotypes in cell density, mutation rate, and the number of potential mutations that confer sufficient resistance to allow growth at a given drug concentration (i.e., the effective mutational target size). To examine these factors, we performed Luria-Delbrück fluctuation tests [48,54,55] with the LTEE ancestor and 2,000-generation clone 2A at tetracycline concentrations of 4 μg/mL and 2 μg/mL, respectively. We used these different antibiotic concentrations because, in our evolvability assays, the 2,000-generation clone had never produced any mutants that formed colonies at 4 μg/mL (Fig 4). We grew 96 replicate 0.1-mL cultures of each strain, starting from small population sizes to ensure mutational independence [48,55]. Because antibiotic-resistant mutants often grow more slowly than their progenitors [20,22–24,26,30], we used the “p_0_” method to estimate effective mutation rates. This method is insensitive to possible differences in growth rate between parent strains and daughter mutants, as it uses only the fraction of the replicate assays that do not yield any mutants [48,55]. For each strain, we used 12 cultures to estimate the population size and 84 cultures to test for resistant mutants at the relevant concentration.

The 2,000-generation clone yielded only about half the cell density as the ancestral strain (8.4 × 10^7^ versus 1.8 × 10^8^ cells per 0.1-mL culture, *p* < 0.0001, based on a two-tailed Welch’s *t* test), which indicates one factor that would contribute to its lower evolvability. For the ancestral strain, 11/84 test cultures yielded at least one mutant resistant to 4 μg/mL, while the other 73 cultures yielded none. For the derived strain, 26 test cultures yielded one or more mutants resistant to 2 μg/mL, while 58 cultures produced none. Using the p_0_ method, the estimated effective mutation rate for the ancestral strain is 7.7 × 10^−10^ per cell generation (approximate 95% confidence limits of 4.2 × 10^−10^ − 1.4 × 10^−9^ based on the uncertainty in p_0_ only [56]), and for clone 2A the estimated rate is 4.4 × 10^−9^ (approximate 95% confidence limits of 3.0 − 6.4 × 10^−9^).

At first glance, it may seem counterintuitive that the estimated mutation rate was higher for the derived clone than for the ancestor, but recall that we tested this clone at a lower antibiotic concentration. Hence, the two rates are not directly comparable. Moreover, published genome sequences and analyses [34] indicate that the underlying point-mutation rate in clone 2A is the same as the ancestral rate. The effective mutation rate that is estimated using a fluctuation test depends on the product of the underlying mutation rate and the effective mutational target size (i.e., the number of mutations that would allow a colony to grow on the antibiotic test plate). Given the same underlying mutation rate, the difference in the estimated mutation rates implies that the derived clone has a larger mutational target at 2 μg/mL of tetracycline than the ancestral strain has at 4 μg/mL of that antibiotic. These analyses indicate that differences in evolvability between genotypes may reflect differences in several factors—cell density, underlying mutation rate, and effective target size—that depend not only on the genetic background but also reflect complex interactions between the genetic background, potential resistance mutations, and the selective environment.

## Discussion

In this study, we addressed a fundamental question about how relaxed selection on a particular set of organismal traits affects their evolvability in situations in which those traits again become advantageous. The traits we studied are resistances to several antibiotics, and the question of how changes in genetic background that occur during relaxed selection affect the subsequent evolvability of resistance has potentially important implications for public health. To address these issues, we examined the capacity of *E*. *coli* strains to evolve increased resistance to four different antibiotics after they had evolved in a drug-free environment for 50,000 generations as part of the LTEE.

We confirmed that intrinsic resistance tended to decay among the LTEE-derived clones to all four antibiotics we tested (Fig 2, Table 1). Our results are consistent with a recent study that examined losses of intrinsic resistance in all 12 LTEE lines at generations 2,000 and 50,000 [32]. Unlike that previous work, however, we then also examined whether and how the LTEE-derived bacteria had changed in their evolvability, specifically their potential to evolve resistance when challenged across a range of concentrations of the same four antibiotics.

We examined two alternative hypotheses that might bear on resistance evolvability. The first is called diminishing returns (Fig 1B), and it often characterizes the course of adaptive evolution [30,37,38,42,46,57,58]. For example, one study used rifampicin-resistant mutants to examine the relation between their initial fitness costs in the absence of this drug and their ability to reduce or eliminate those costs during subsequent evolution, again in a drug-free environment [30]. They first isolated eight *rpoB* mutants after a single round of antibiotic selection and showed that the mutants varied in their fitness defects. The authors then propagated these mutants and detected the first beneficial mutations to sweep to high frequency in those populations. They found that the lower-fitness backgrounds gave rise to mutations that conferred greater advantages than did the backgrounds that initially had higher fitness, in accordance with a diminishing-returns model.

If the evolution of antibiotic resistance after a period of decay under relaxed selection conformed to the diminishing-returns model, then we would expect the more susceptible LTEE-derived backgrounds to be more evolvable than their common ancestor. However, we found little statistical support for diminishing returns in our study (Fig 3, Table 2). There was one instance in which an individual LTEE-derived clone was significantly more evolvable than the ancestor in the ampicillin environment, and two other clones trended in this direction (Fig 3A, S2 Table). However, the statistical support, even for ampicillin, was marginal at best when the evolvabilities of the four clones were analyzed together to account for multiple tests of the same hypothesis (Table 2). In any case, diminishing returns was not typical across the entire set of experiments.

The absence of an overall trend toward diminishing returns might be attributable in part to two methodological issues. First, it might point to a limitation of our plate-based approach, and conventional MIC assays in general, to discern subtle differences in MICs and hence in evolvabilities based on differences in MIC values. That is, slight differences in evolvability may be obscured by the discrete resolution of the assays using 2-fold increasing concentrations of an antibiotic. Consistent with this possibility, the range of initial susceptibilities was greatest in the ampicillin environment, where the trend toward diminishing returns was most evident (Fig 3A). An alternative approach that might better capture subtle trends would be to use a continuous culture device that dynamically adjusts drug concentration in the growth medium to match the ongoing adaptive dynamics of the population under study [49]. Second, we might have had insufficient statistical power to resolve diminishing-returns trends in evolvability. We tested four LTEE-derived lines and their ancestor, whereas some other studies that show diminishing returns in other contexts have used as many as hundreds of lines [38].

There is a third factor—one that is biological, rather than methodological—that could also obscure any tendency toward diminishing returns, and that is idiosyncratic heterogeneity among genetic backgrounds in their evolvability (Fig 1C). This pattern occurs when particular mutations that arose during relaxed selection happen to either constrain or potentiate a strain’s future evolutionary potential with respect to a given selective pressure. We found that the capacity to evolve ceftriaxone resistance tended to be lower for the derived clones than for the ancestor (Fig 3B, Table 3). That tendency is in contrast to the ampicillin environment, and it is unexpected given that both drugs are β-lactams that target cell-wall synthesis. In a similar vein, we also demonstrated that the Ara+5 lineage had become significantly constrained in its ability to evolve tetracycline resistance relative to both its ancestor and the other LTEE-derived lineages (Fig 3D).

We conclude, therefore, that historical contingency has played an important role in the capacity of the LTEE-derived populations to respond evolutionarily to changed environments, in particular when challenged with antibiotics. That is, different lineages accumulated genetic differences—even in replicate populations that evolved in the same environment—that influence their ability to evolve and adapt in new directions. Several other microbial evolution studies have also documented cases of historically contingent outcomes. For example, the mutations that accumulated in one LTEE population potentiated the subsequent evolution of a novel metabolic capacity that arose in only that one population, despite comparable time and opportunity in the 11 other replicate populations [39,59,60]. Similarly, an experiment with *Pseudomonas aeruginosa* showed that high-level colistin resistance was potentiated by prior mutations in transcriptional regulators *phoQ* and *pmrB* [61]. However, the consequences of contingency in these two cases were the opposite of what we saw in our study: namely, evolvability was potentiated in these studies, whereas it became more constrained in ours. Still other studies have found little evidence for historical contingencies affecting evolvability. For example, Travisano and colleagues [36] isolated a clone from each LTEE population after 2,000 generations in the glucose-limited medium. They then founded three replicate populations from each clone and let them evolve for 1,000 generations in the same environment, except with maltose replacing glucose as the limiting resource. The founding clones had independent histories in the glucose environment, and they varied greatly in their initial fitness in the maltose environment. Despite this initial heterogeneity, however, the populations rapidly converged on similar fitnesses in the new maltose environment. Thus, adaptation dominated over contingency in that experiment.

Returning to our study, we ask the following questions: When did the evolvability with respect to tetracycline exposure decline in the Ara+5 population? And why, in molecular-genetic terms, did it decline? To answer the first question, we tested clones from throughout this population’s history and identified when the bacteria first lost their ability to evolve resistance to the same degree as the ancestral strain. We found that this constraint was already present in one of two clones sampled at 1,500 generations of the LTEE, and it was evident in all of the clones we tested from generation 2,000 and onward (Table 4) despite these backgrounds having retained the same initial resistance level as the ancestor (Fig 4). These data thus confirm the idiosyncratic effects of genetic background on the evolvability of resistance. By performing fluctuation tests to measure effective mutation rates, we also showed that evolvability can depend in complex ways on several factors, including not only a strain’s underlying mutation rate but also the cell density it achieves and the effective mutational target size for a given antibiotic concentration.

With respect to the second question, we do not yet know the answer, but the early timing of the change in evolvability allows us to narrow substantially the genetic possibilities. By combining our phenotypic results with previously obtained genomic data [34], we have identified three candidate mutations that alone or in combination could explain this reduced evolvability. These mutations arose in the following genes: *mreB*, which encodes a protein involved in cell-wall structuring; *pykF*, which encodes pyruvate kinase that catalyzes the last step of glycolysis; and *trkH*, which encodes a potassium ion transporter. Interestingly, recent studies have discovered that mutations in *trkH* can cause increased susceptibility to tetracycline through changes to the proton-motive force, and this relationship may depend upon the genetic background [62,63]. In future work, we hope to make genetic constructs that will allow us to investigate the genetic basis for the low evolutionary potential of this background when exposed to tetracycline. We will also sequence some of the antibiotic-resistant mutants that evolved in our experiments to test whether there are any systematic differences among the various strains in the genetic targets of the resistant mutations and whether such differences correlate with the history of relaxed selection and concomitant increased susceptibility.

In summary, we have shown that bacterial evolution in the absence of antibiotic exposure can lead not only to increased susceptibility but also to genetic background–dependent changes in resistance evolvability when cells are exposed to those drugs. The evolution of resistance can thus depend upon previously accumulated mutations in a historically contingent fashion. These findings could have important health implications if evolution in an antibiotic-free environment sometimes erodes not only a pathogen’s resistance level but also its potential to evolve greater resistance. We therefore suggest that strategic antibiotic management may benefit not only from surveillance of current resistance levels in pathogens but also from analyses of their potential to evolve increased resistance. This approach could be valuable both on the scale of the individual patient, where effective treatment is paramount, and on a community-wide scale, where judicious efforts to control the spread of drug resistance become critical. We hope that our evolvability-based approach and extensions thereof prove useful in achieving these objectives.

## Materials and methods

### Bacterial strains

All of the strains used in this study are from the *E*. *coli* LTEE. In the LTEE, 12 replicate populations were founded from a common ancestral strain called REL606 [33]. These populations have been propagated for over 31 years by daily 1:100 transfers in glucose-supplemented Davis minimal (DM) medium without any antibiotics [33], resulting in >72,000 cell generations to date. Samples from each population are frozen periodically at −80°C. In this study, we quantified the intrinsic antibiotic resistance and evolvability of the ancestor and derived clones isolated from four populations (designated Ara–5, Ara–6, Ara+4, and Ara+5) after 50,000 generations of the LTEE. We chose these strains for two reasons. First, the source populations of these derived clones retained the low ancestral mutation rate, and therefore they accumulated many fewer mutations than their counterparts from several populations that evolved hypermutability [34]. This characteristic should increase the tractability of identifying candidate alleles affecting resistance evolvability, which we hope to achieve in future work. Second, generation 50,000 is the latest point at which whole-genome sequence data are available for the clonal samples [34].

We also examined when the Ara+5 population evolved a diminished capacity to increase its tetracycline resistance (as described in the Results) by testing two strains isolated from this population at several earlier time points (generations 500, 1,000, 1,500, 2,000, 5,000, and 10,000). All of the strains used in this study are listed in S3 Table.

### Culture conditions and measurements of resistance and evolvability

All experiments were performed at 37°C. Bacterial strains were revived from frozen stocks by overnight growth in Luria Bertani (LB) medium. Cells from these cultures were then streaked onto DM agar plates supplemented with 4 mg/mL glucose. We randomly picked single isolated colonies from these plates to start multiple replicate populations in LB. Final population sizes in the LB cultures were approximately 1 − 2 × 10^9^ cells/mL. When an initially susceptible cell expands into a colony and then a population, new mutations spontaneously occur and increase in number during growth [48]. The evolution of antibiotic-resistant mutants will therefore originate by independent mutational events in each replicate population [48,64].

We define a strain’s evolvability as the maximum increase in antibiotic resistance from an initially susceptible genotype during one round of drug selection. Evolvability experiments were performed using Mueller-Hinton (MH) agar (Acumedia, Lansing, MI) supplemented with 1 mg/mL glucose, 0.1 mg/mL magnesium sulfate, 0.01 mg/mL thiamine, and a series of 2-fold dilutions of an antibiotic. We used MH agar because a previous study used this medium to quantify the susceptibilities of LTEE clones to various antibiotics [32]. Our study, in part, sought to replicate these findings. We chose the four antibiotics in our study because they have diverse cellular targets: ampicillin and ceftriaxone inhibit cell-wall synthesis, ciprofloxacin inhibits DNA replication, and tetracycline inhibits protein synthesis. We prepared stock solutions of each antibiotic following the manufacturers’ instructions, which were then stored at −20°C.

One-milliliter samples of each population were centrifuged at 8,000 rpm for 2 minutes and resuspended in an equal volume of saline. We then plated 100 μL (containing approximately 1 − 2 × 10^8^ cells) from each suspension onto the antibiotic-amended MH agar plates, and MICs were evaluated after 48 hours of incubation. For this study, we operationally define a pair of MICs for each series of antibiotic-amended plates as the lowest concentration that prevents either confluent growth or isolated colonies. According to this approach, confluence indicates growth by the susceptible “parental” strain, while isolated colonies are resistant “daughter” mutants. A strain’s evolvability was calculated from the difference in MIC between these two genotypes. For each experimental block, putative resistant mutants were confirmed by streaking one randomly chosen colony per strain onto fresh antibiotic-amended MH plates. All clones regrew at the corresponding concentration. This approach indicated that a selected clone was indeed a resistant mutant with a stably inherited increase in its MIC, as opposed to a so-called “persister” that exhibited higher-than-average phenotypic tolerance relative to genetically identical cells [65]. Cultures of mutant clones were then frozen at −80°C in LB medium supplemented with 15% glycerol as a cryoprotectant.

S1 Fig provides a schematic representation of our methods for measuring the MICs of sensitive parental strains and their resistant daughter derivatives. In S2 Fig, we show an image of the resulting plates for one replicate series across a 256-fold (= 2^8^) range of ciprofloxacin concentrations for the LTEE ancestral clone. In this image, one sees confluent growth on the first 3 plates, isolated colonies on the next 2 plates, and no evident growth on the 4 plates with the highest concentrations. Based on these plates, we scored the MIC of the sensitive parental strain as the lowest concentration that inhibited confluent growth, which was 0.0025 μg/mL in this example. We scored the MIC of the resistant daughter derivative as the lowest concentration where even isolated colonies were absent, in this case 0.01 μg/mL. The log_2_-transformed difference between these values (i.e., log_2_ 0.01/0.0025 = 2 in this example) provides one estimate of the evolvability of the LTEE ancestral strain with respect to ciprofloxacin. We obtained 32 independent estimates of these MICs and the associated evolvabilities for the ancestral strain against each of the four antibiotics used in our study. We similarly obtained eight independent estimates of the MICs and associated evolvabilities for each of the four 50,000-generation strains used in our study against each of the same antibiotics. Photographs of all of the replicate plate series used to estimate these values have been archived on the Dryad Digital Repository: https://datadryad.org/stash/dataset/doi:10.5061/dryad.g41hg96 [66].

### Experimental design and data analyses

All MIC values were transformed by taking their base-2 logarithm because the antibiotic concentrations were tested across a series of 2-fold dilutions. For each experimental block, an independently isolated LTEE ancestral clone was paired with each derived clone. We had two predictions when we began this study: (i) the derived bacteria would be more susceptible to antibiotics (lower MICs) than their common ancestor as a consequence of the relaxed selection they experienced in the permissive LTEE environment and (ii) the derived bacteria would be more evolvable than their ancestor when challenged with antibiotics, following a general trend of diminishing-returns adaptation.

Statistical tests that rely on normally distributed data were deemed inappropriate for this study owing to the discrete, lumpy nature of the measurements. Instead, we used nonparametric methods. There were also numerous instances in which the derived clones were equal both in MIC and evolvability to the paired assays for the ancestor, and these ties introduced additional complications. Therefore, we used trinomial tests to examine changes in the direction of our expectations relative to the null hypothesis that changes are equally frequent in either direction [51]. We performed these analyses by individually comparing the four derived clones with their paired ancestors across each antibiotic. Probabilities were then combined from these independent significance tests using Fisher’s method with 8 degrees of freedom (i.e., df = 2*k*; where *k* is the number of comparisons) [52,53]. As explained previously (Fig 1C), evolvability might be idiosyncratic and therefore not correlated with the initial level of resistance. To assess this possibility, we performed a Kruskal-Wallis one-way ANOVA to test for heterogeneity in evolvability among the LTEE lines. Datasets and the details of our statistical analyses are provided in an R Notebook on the Dryad Digital Repository: https://datadryad.org/stash/dataset/doi:10.5061/dryad.g41hg96 [66].

We performed fluctuation tests to estimate effective mutation rates [48] for the ancestral strain (REL606) to 4 μg/mL tetracycline, and for a derived clone from the Ara+5 population (REL1162A) to 2 μg/mL tetracycline. The two strains were taken from the freezer and grown overnight in LB broth. Each culture was then serially diluted in saline solution, and fewer than 1,000 cells were transferred into each well of a 96-well plate; each well contained 0.1 mL of LB broth. After 24 hours, we removed the entire volume from each of 84 wells and spread it on an MH agar plate amended with either 2 μg/mL or 4 μg/mL tetracycline for REL1162A or REL606, respectively. The other 12 wells were sampled to enumerate the bacteria using a Coulter counter (Multisizer 4e, Beckman) with a 30-μm aperture; we set a cutoff of 0.2 fL to distinguish cells from background debris and subtracted counts from a sterile LB-only negative control. We incubated the antibiotic-amended plates for 48 hours and we then scored each plate for the absence or presence of one or more colonies, as required for the p_0_ method to estimate mutation rates.

## Supporting information

S1 Fig Schematic illustration of the LTEE and evolvability study design. (A) Twelve initially identical *E*. *coli* populations were founded from a common ancestor to start the LTEE. These populations have evolved for >72,000 generations with daily serial transfers in a minimal medium without antibiotics. (B) In this study, antibiotic-susceptible ancestral or derived clones from generation 50,000 were inoculated into replicate cultures. A resistance mutation may arise spontaneously and increase in number during a population’s expansion, resulting in two genetic variants: the susceptible parental cells and their descendent resistant daughters. (C) These whole populations were then spread onto agar plates supplemented with 2-fold increasing concentrations of an antibiotic (shown in red). MICs of these two variants correspond to the lowest antibiotic concentration that inhibits confluent growth and that prevents even isolated colonies, respectively. Resistant clones were confirmed by streaking onto fresh plates with relevant antibiotic concentrations. LTEE, long-term evolution experiment; MIC, minimum inhibitory concentration.
(TIF)

S2 Fig Experimental plates. Whole populations containing susceptible parental and resistant daughter cells were spread onto MH agar amended with 2-fold increasing concentrations of ciprofloxacin (left to right, and down). Confluent lawns of bacterial growth (plates 1–3) consist largely of drug-susceptible cells. Isolated colonies (plates 4–5) are putatively resistant mutants. Images of all experimental plates have been archived on the Dryad Digital Repository: https://datadryad.org/stash/dataset/doi:10.5061/dryad.g41hg96. MH, Mueller-Hinton.
(TIF)

S1 Table Statistical significance for changes in intrinsic resistance of the individual clones sampled at generation 50,000 of the LTEE for the four antibiotic treatments. Analyses were performed based on a trinomial distribution, which reflects the many ties in these datasets. The reported *p*-values are one-tailed, which reflects the expectation that resistance should decline under relaxed selection in the antibiotic-free LTEE environment. LTEE, long-term evolution experiment.
(DOCX)

S2 Table Statistical significance for trends of diminishing-returns resistance evolvability of the individual clones sampled at generation 50,000 of the LTEE for the four antibiotic treatments. Analyses were performed based on a trinomial distribution, which reflects the many ties in these datasets. The reported *p*-values are one-tailed, which reflects the directional expectation implied by diminishing returns. LTEE, long-term evolution experiment.
(DOCX)

S3 Table Bacterial strains used in this study. All clones were derived from REL606, the ancestral strain of the LTEE. LTEE, long-term evolution experiment.
(DOCX)

## Abbreviations

DM: Davis minimal
LB: Luria Bertani
LTEE: long-term evolution experiment
MH: Mueller-Hinton
MIC: minimum inhibitory concentration
