# Supplementary figures and images for: Historical contingency in the evolution of antibiotic resistance after decades of relaxed selection
Source: PLoS Biol. 2019 Oct 23;17(10):e3000397. doi: 10.1371/journal.pbio.3000397 (PMC6827916; doi:10.1371/journal.pbio.3000397)

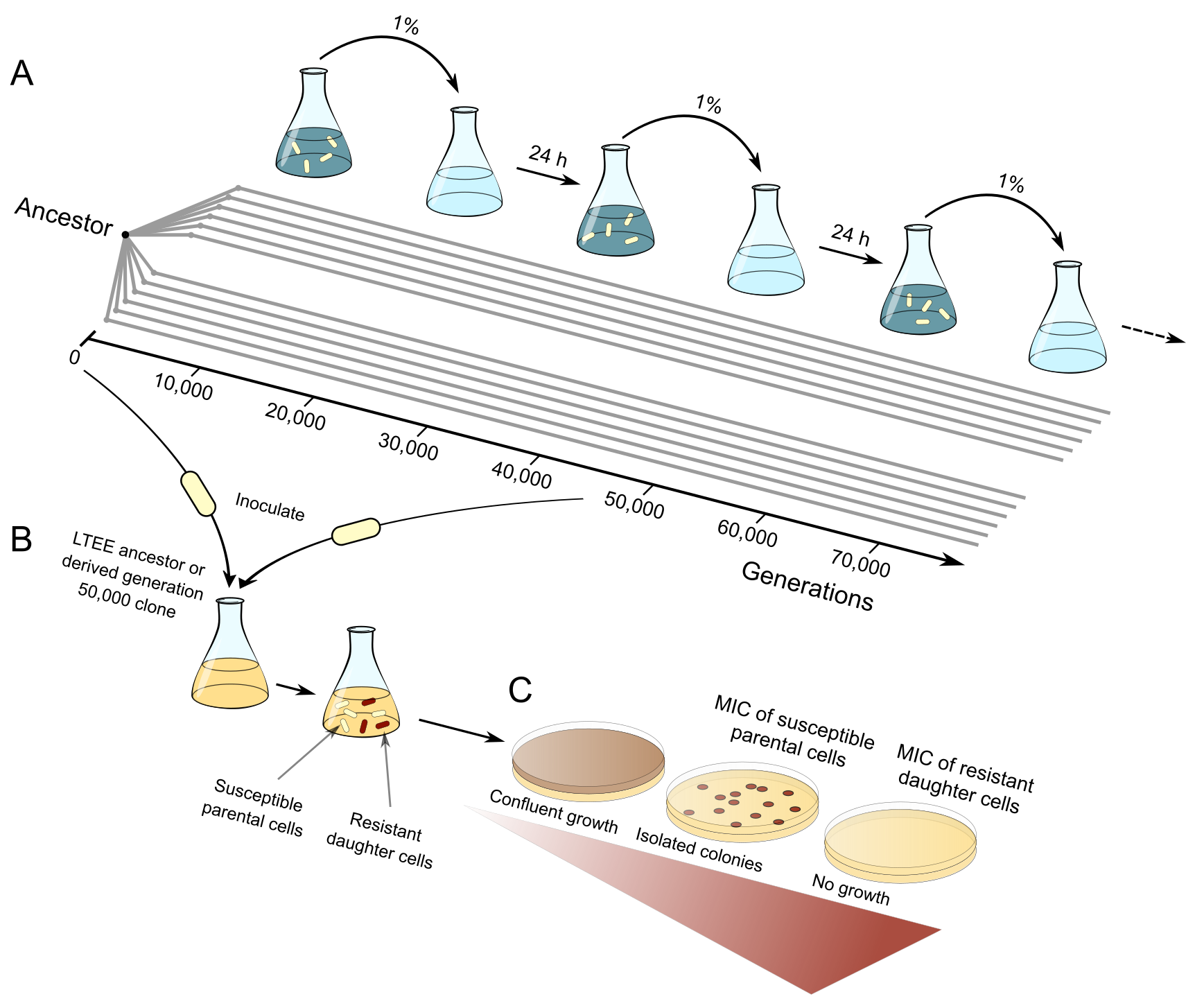

Supplement: S1 Fig — (A) Twelve initially identical E. coli populations were founded from a common ancestor to start the LTEE. These populations have evolved for >72,000 generations with daily serial transfers in a minimal medium without antibiotics. (B) In this study, antibiotic-susceptible ancestral or derived clones from generation 50,000 were inoculated into replicate cultures. A resistance mutation may arise spontaneously and increase in number during a population’s expansion, resulting in two genetic variants: the susceptible parental cells and their descendent resistant daughters. (C) These whole populations were then spread onto agar plates supplemented with 2-fold increasing concentrations of an antibiotic (shown in red). MICs of these two variants correspond to the lowest antibiotic concentration that inhibits confluent growth and that prevents even isolated colonies, respectively. Resistant clones were confirmed by streaking onto fresh plates with relevant antibiotic concentrations. LTEE, long-term evolution experiment; MIC, minimum inhibitory concentration. (TIF) [file pbio.3000397.s001.tif]

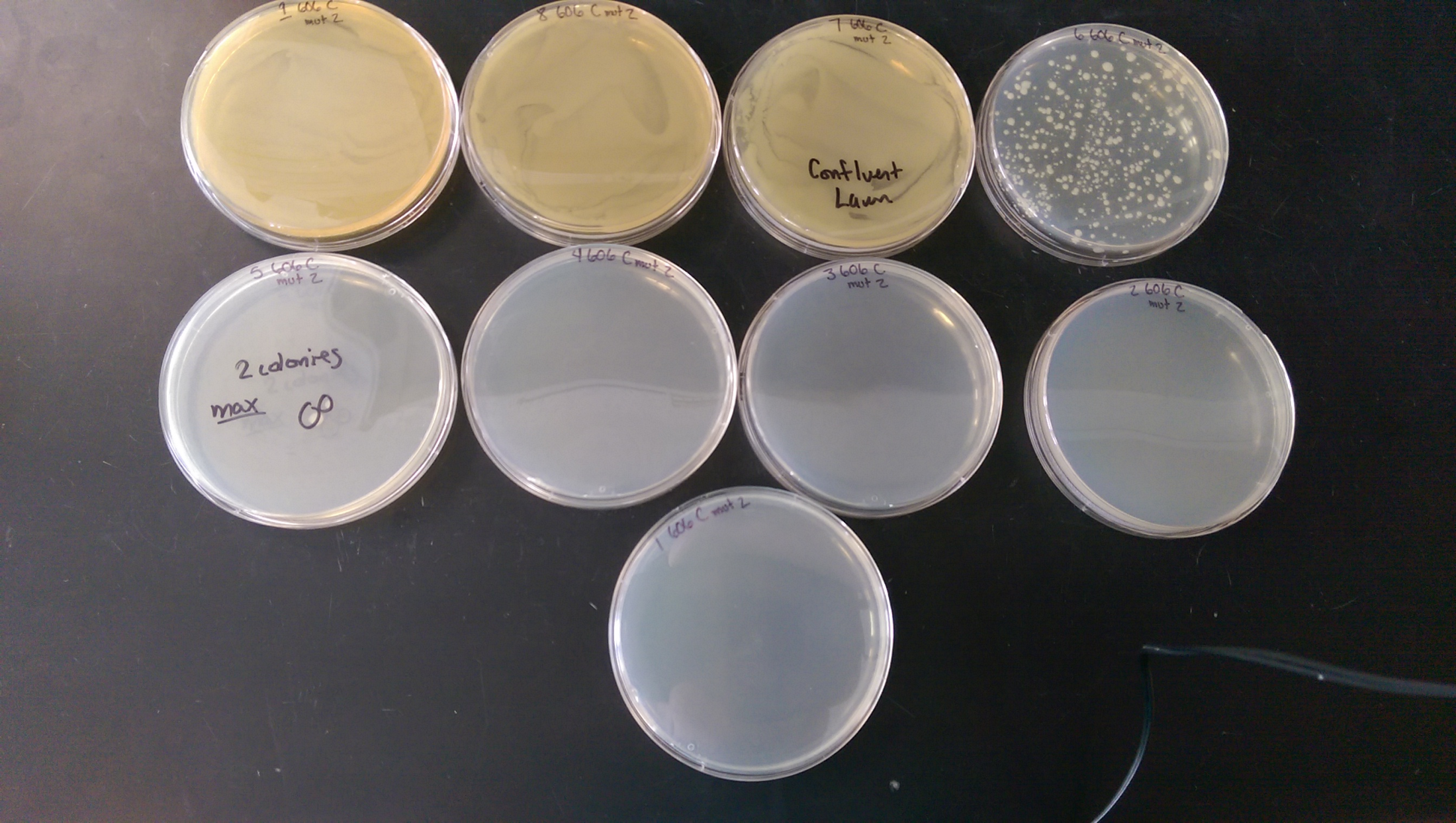

Supplement: S2 Fig — Whole populations containing susceptible parental and resistant daughter cells were spread onto MH agar amended with 2-fold increasing concentrations of ciprofloxacin (left to right, and down). Confluent lawns of bacterial growth (plates 1–3) consist largely of drug-susceptible cells. Isolated colonies (plates 4–5) are putatively resistant mutants. Images of all experimental plates have been archived on the Dryad Digital Repository: https://datadryad.org/stash/dataset/doi:10.5061/dryad.g41hg96. MH, Mueller-Hinton. (TIF) [file pbio.3000397.s002.tif]
