# Supplementary material for: Historical contingency in the evolution of antibiotic resistance after decades of relaxed selection
Source: PLoS Biol. 2019 Oct 23;17(10):e3000397. doi: 10.1371/journal.pbio.3000397 (PMC6827916; doi:10.1371/journal.pbio.3000397)
Supplement: S1 Table — Analyses were performed based on a trinomial distribution, which reflects the many ties in these datasets. The reported p-values are one-tailed, which reflects the expectation that resistance should decline under relaxed selection in the antibiotic-free LTEE environment. LTEE, long-term evolution experiment. (DOCX) [file pbio.3000397.s003.docx]

| **Antibiotic** | **Clone** | ***p*** |
| --- | --- | --- |
| Ampicillin | Ara–5 | 0.0080 |
|  | Ara–6 | 0.0039 |
|  | Ara+4 | 0.0080 |
|  | Ara+5 | 0.0080 |
| Ceftriaxone | Ara–5 | 0.0031 |
|  | Ara–6 | 0.0031 |
|  | Ara+4 | 0.0039 |
|  | Ara+5 | 0.0039 |
| Ciprofloxacin | Ara–5 | 0.2177 |
|  | Ara–6 | 0.1004 |
|  | Ara+4 | 0.0149 |
|  | Ara+5 | 0.0039 |
| Tetracycline | Ara–5 | 0.0031 |
|  | Ara–6 | 0.0031 |
|  | Ara+4 | 0.0039 |
|  | Ara+5 | 0.0278 |
