# Supplementary material for: Historical contingency in the evolution of antibiotic resistance after decades of relaxed selection
Source: PLoS Biol. 2019 Oct 23;17(10):e3000397. doi: 10.1371/journal.pbio.3000397 (PMC6827916; doi:10.1371/journal.pbio.3000397)
Supplement: S3 Table — All clones were derived from REL606, the ancestral strain of the LTEE. LTEE, long-term evolution experiment. (DOCX) [file pbio.3000397.s005.docx]

| **Strain** | **Generation** | **LTEE Population** |
| --- | --- | --- |
| REL606 | 0 | - |
| REL772A | 500 | Ara+5 |
| REL772B | 500 | Ara+5 |
| REL962A | 1,000 | Ara+5 |
| REL962B | 1,000 | Ara+5 |
| REL1066A | 1,500 | Ara+5 |
| REL1066B | 1,500 | Ara+5 |
| REL1162A | 2,000 | Ara+5 |
| REL1162B | 2,000 | Ara+5 |
| REL2177A | 5,000 | Ara+5 |
| REL2177B | 5,000 | Ara+5 |
| REL4534A | 10,000 | Ara+5 |
| REL4534B | 10,000 | Ara+5 |
| REL11339 | 50,000 | Ara–5 |
| REL11389 | 50,000 | Ara–6 |
| REL11348 | 50,000 | Ara+4 |
| REL11367 | 50,000 | Ara+5 |
